# Supplementary figures and images for: Differential proteomic analysis under pesticides stress and normal conditions in Bacillus cereus 2D
Source: PLoS One. 2021 Aug 13;16(8):e0253106. doi: 10.1371/journal.pone.0253106 (PMC8362991; doi:10.1371/journal.pone.0253106)

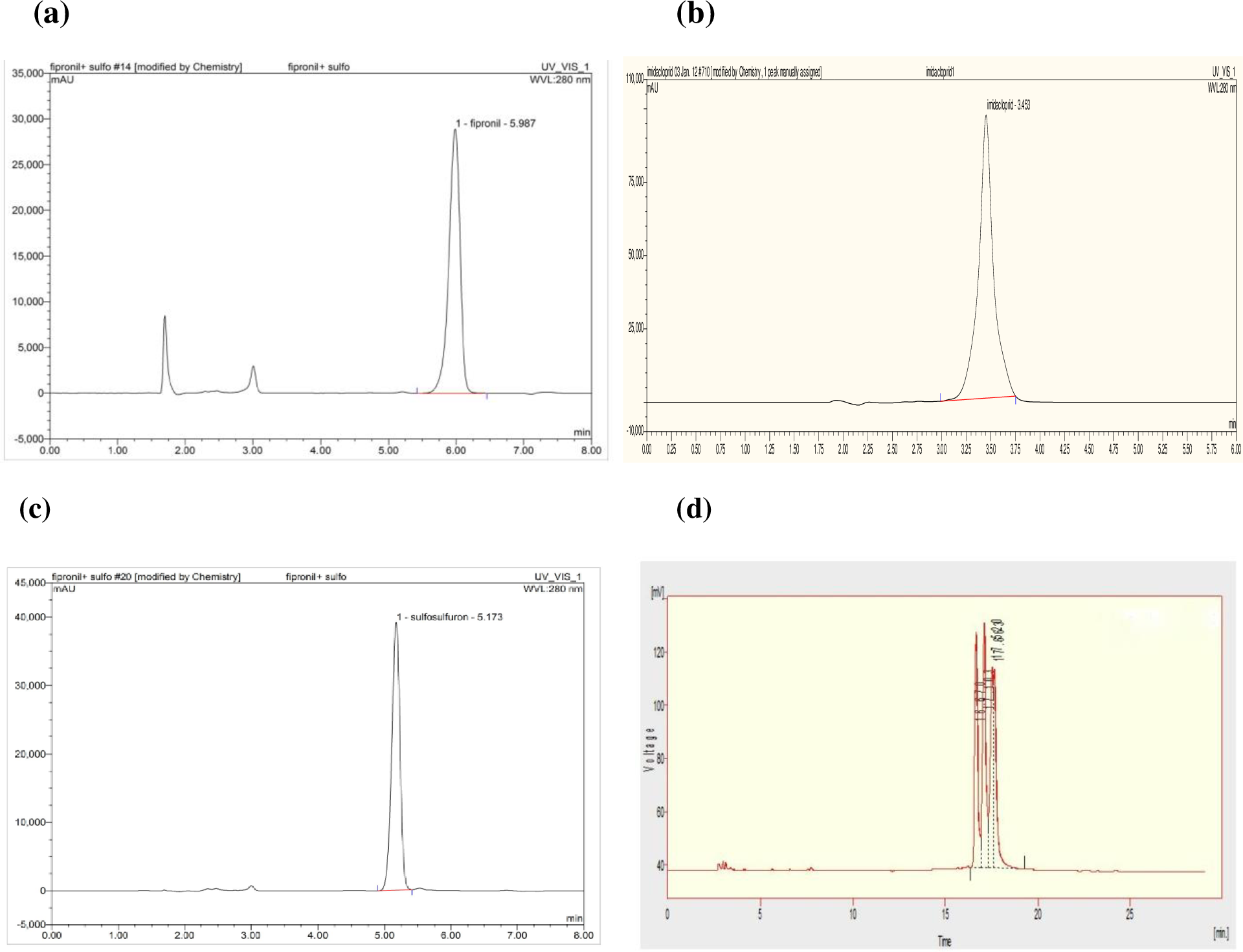

Supplement: S1 Fig — Standard peaks of imidacloprid (a), fipronil(b), sulfosulfuron(c) and cypermethrin(d) at 10 ppm concentration. (TIF) [file pone.0253106.s001.tif]

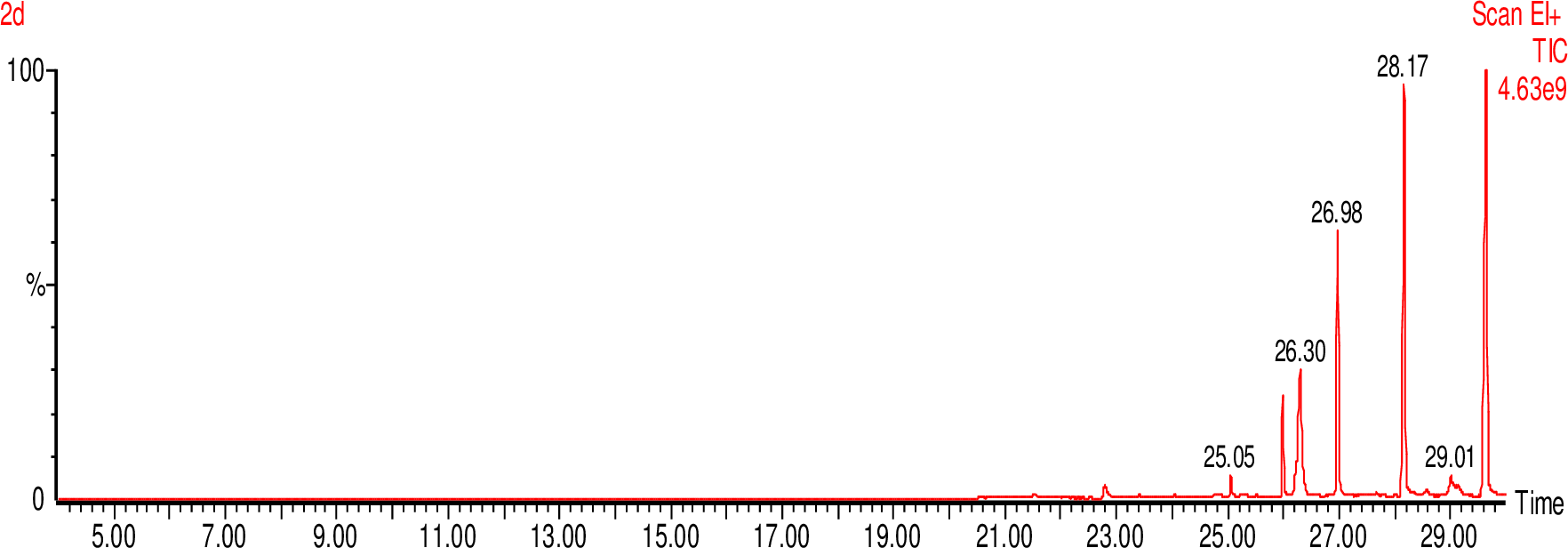

Supplement: S2 Fig — (TIF) [file pone.0253106.s002.tif]

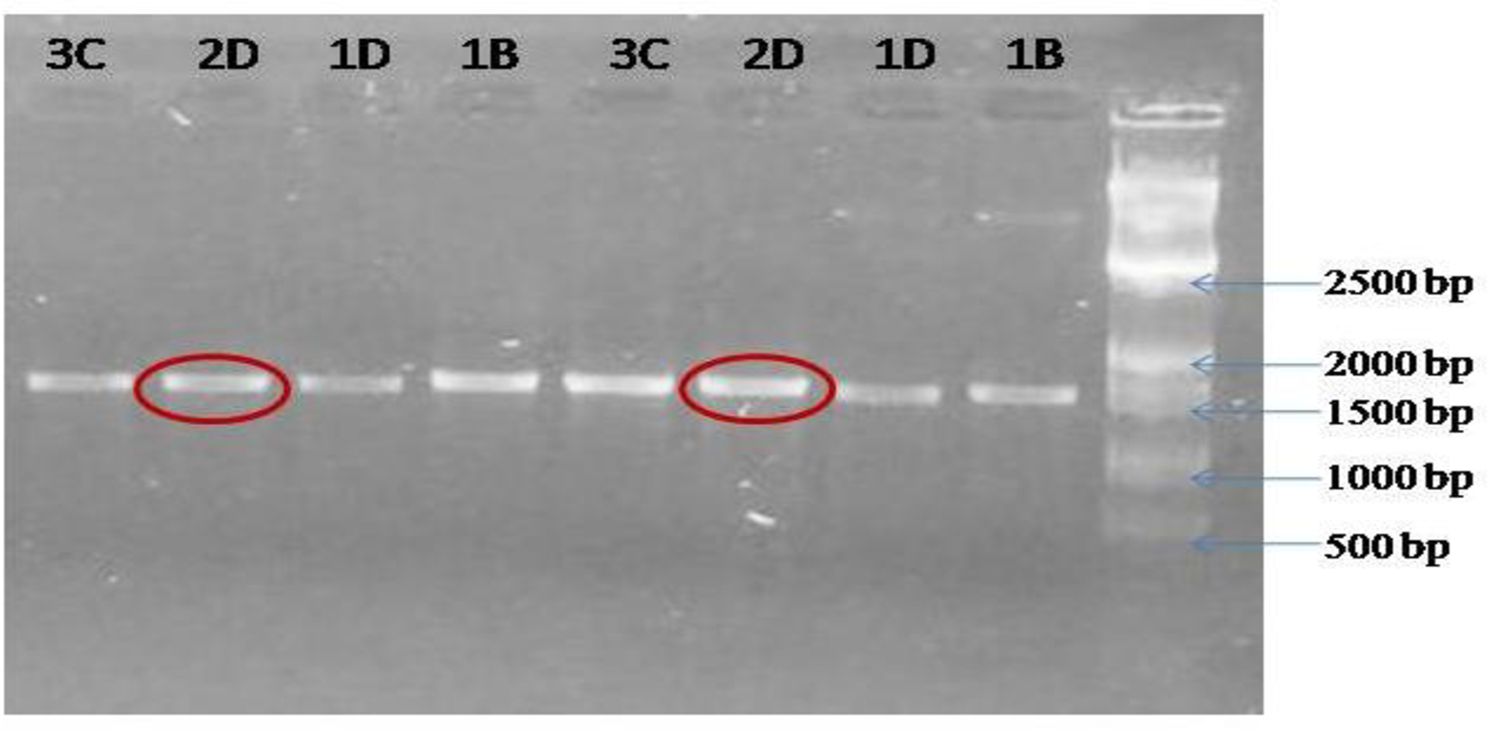

Supplement: S3 Fig — (TIF) [file pone.0253106.s003.tif]

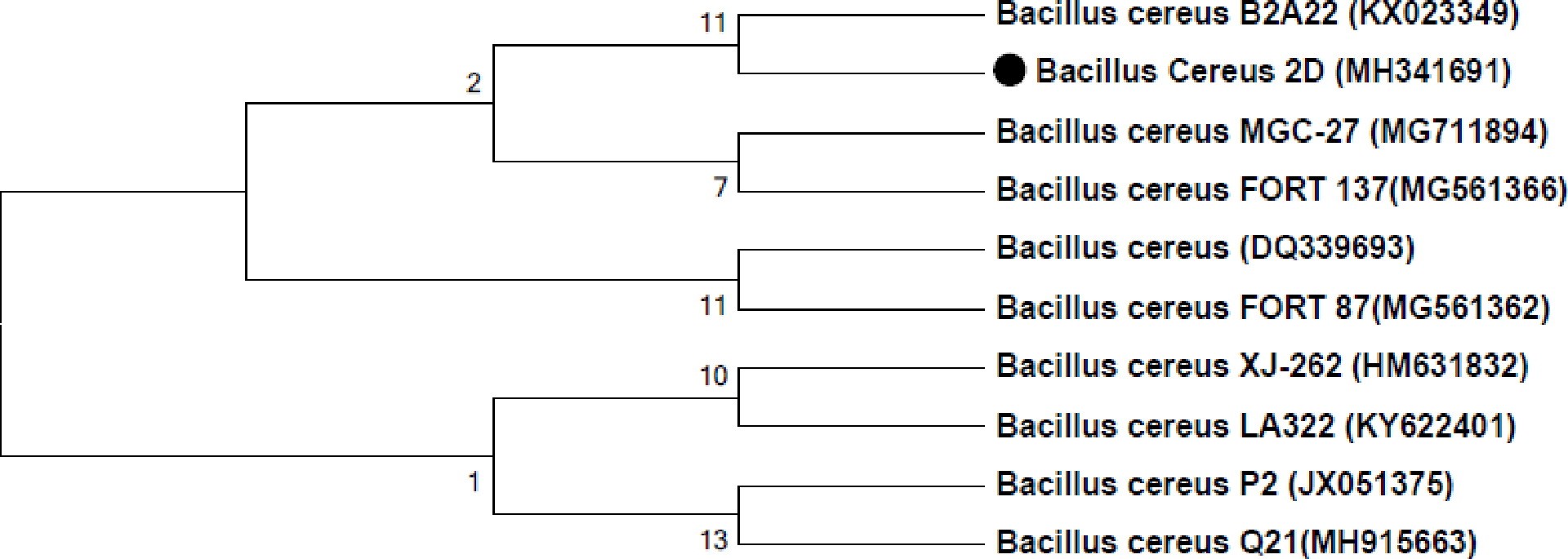

Supplement: S4 Fig — (TIF) [file pone.0253106.s004.tif]

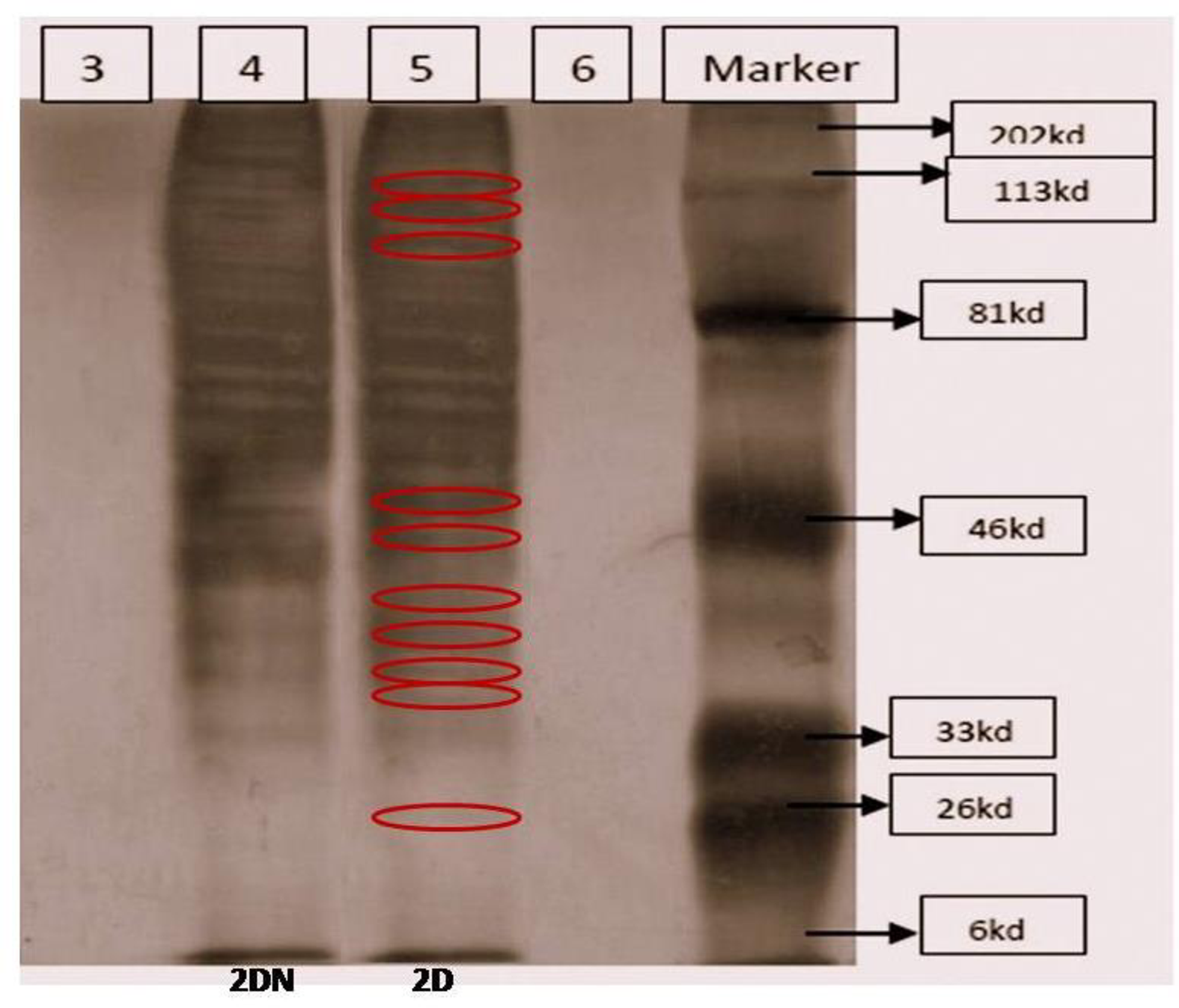

Supplement: S5 Fig — Bacterial strains designated as 2D in lane number 5 and 2DN in lane number 4 respectively under stress and normal condition. The bacterial proteins expressed in stress conditions are denoted by highlighted encircled. (TIF) [file pone.0253106.s005.tif]

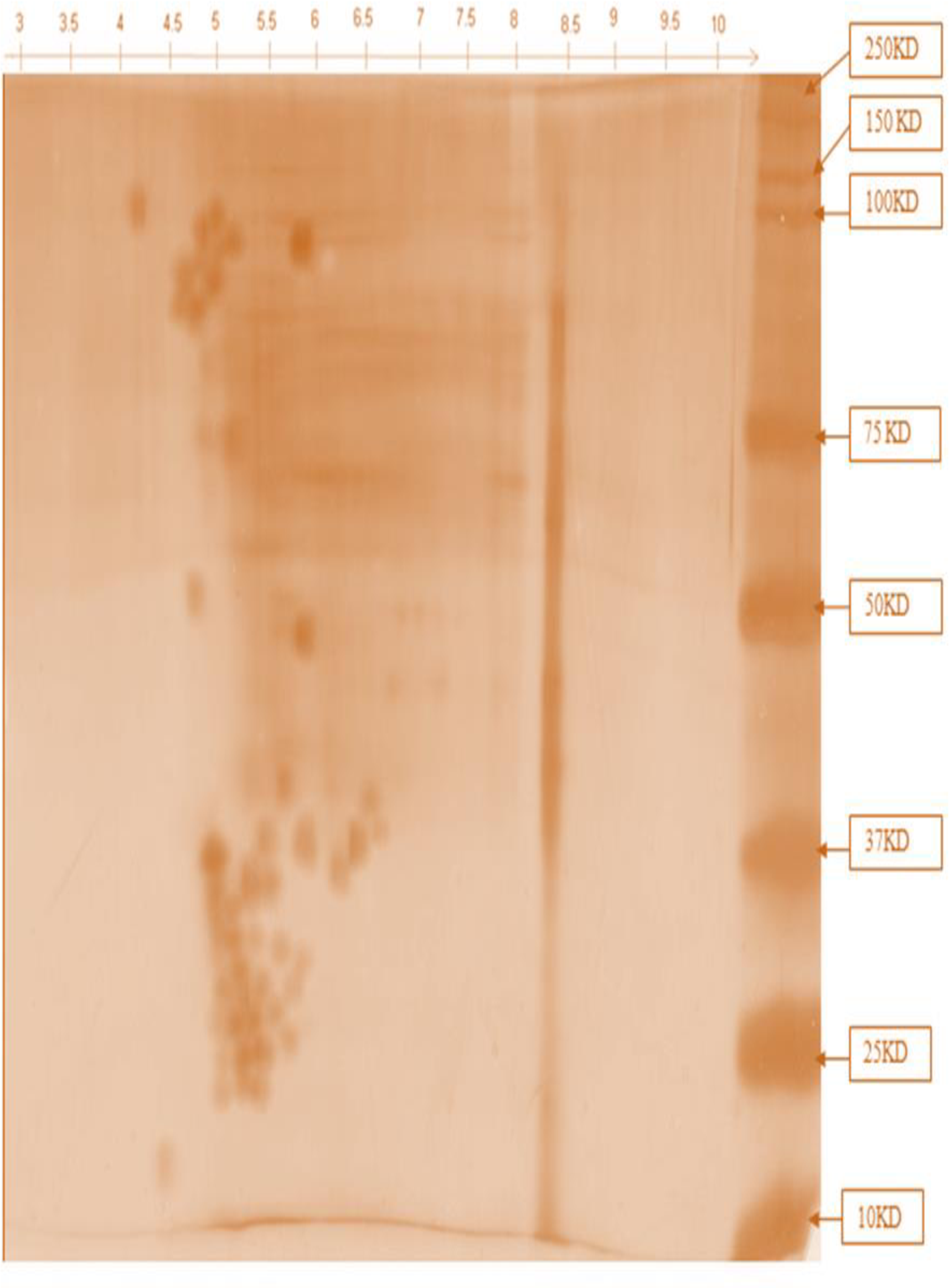

Supplement: S6 Fig — (TIF) [file pone.0253106.s006.tif]

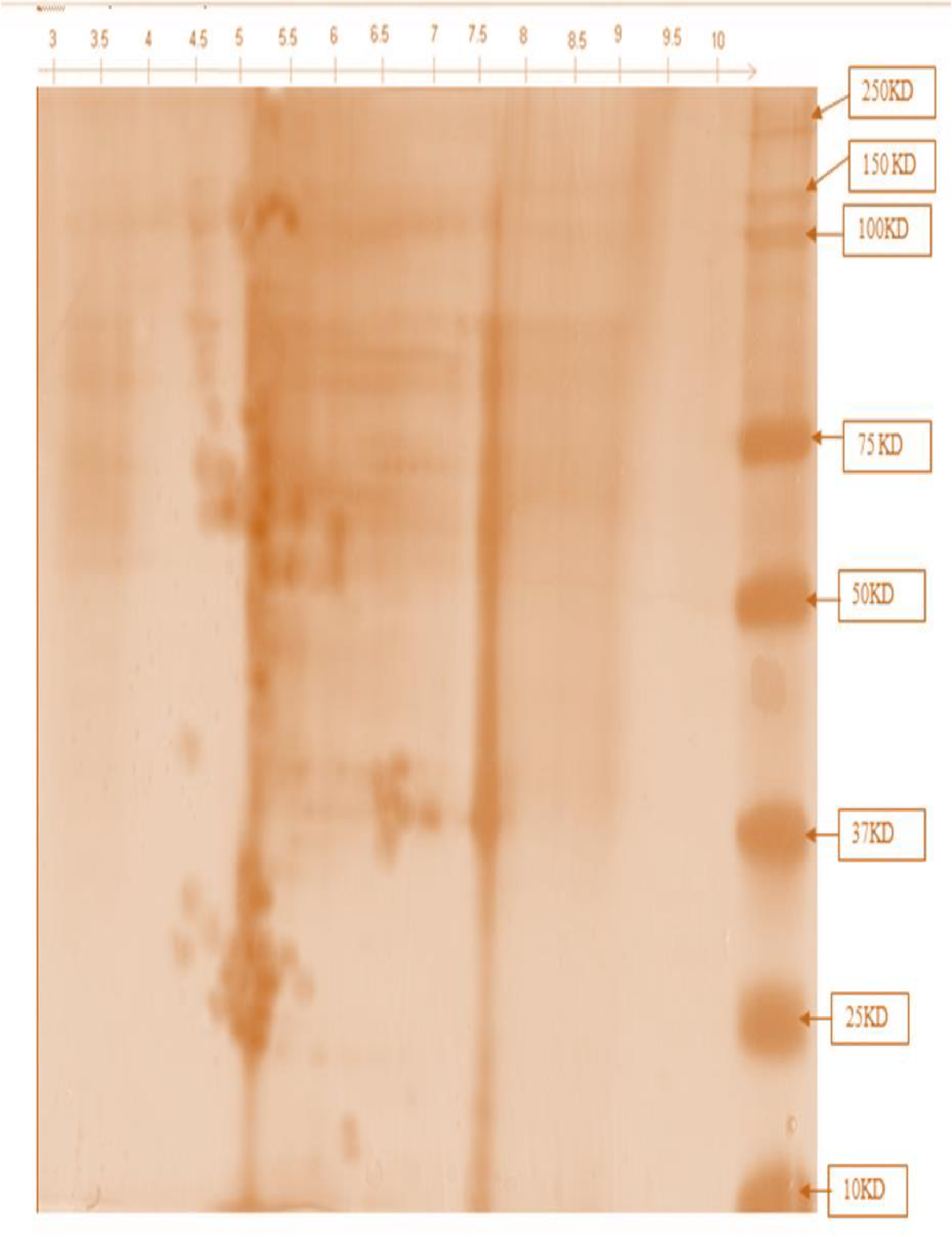

Supplement: S7 Fig — (TIF) [file pone.0253106.s007.tif]
