# Supplementary material for: Differential proteomic analysis under pesticides stress and normal conditions in Bacillus cereus 2D
Source: PLoS One. 2021 Aug 13;16(8):e0253106. doi: 10.1371/journal.pone.0253106 (PMC8362991; doi:10.1371/journal.pone.0253106)
Supplement: S1 File — (DOCX) [file pone.0253106.s008.docx]

**Clarification regarding images**

Dear managing editor all the images of the research article are original. Although to make good representation, some of the images are edited. In support of the figures or images attached in the research articles hereby attached original images(unedited) of the respective figures. The data represented in the paper is from my Ph.D. thesis. The link for data is <https://krishikosh.egranth.ac.in/handle/1/5810096352>. Some of the data were generated by outsourcing and they provided data only in pdf form.


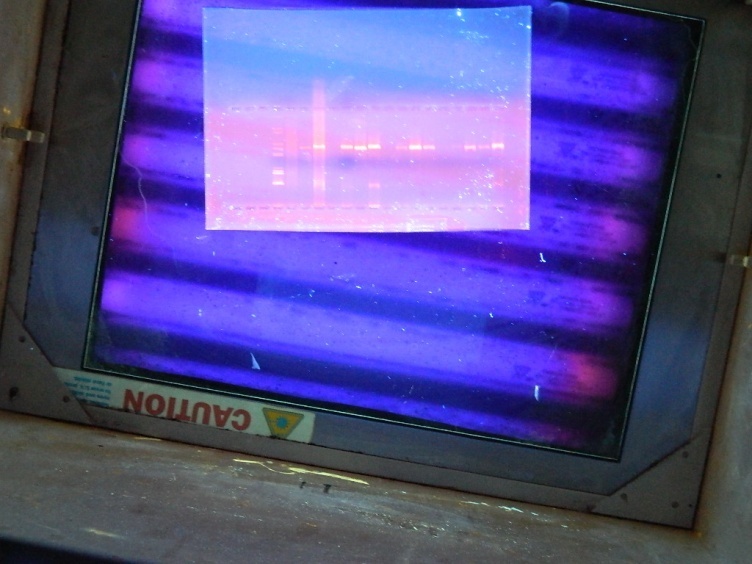


**Fig:** Laccase gel with different bacterial isolates


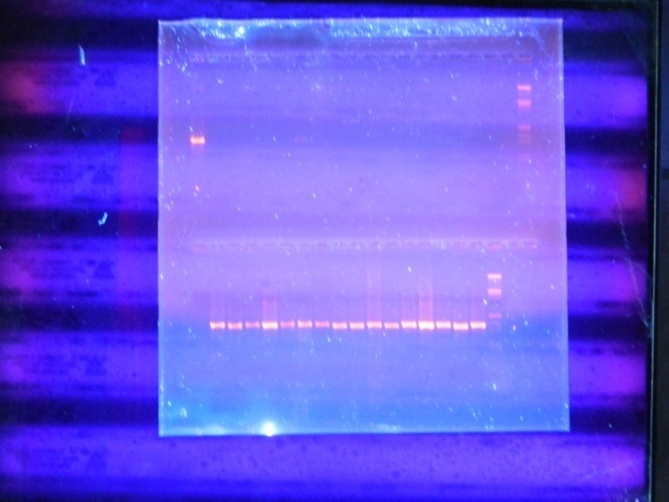


**Fig:** 16S rDNA amplification gel


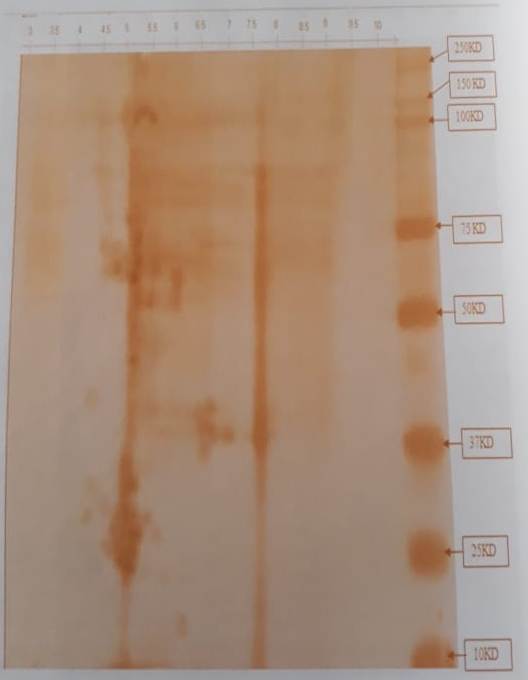


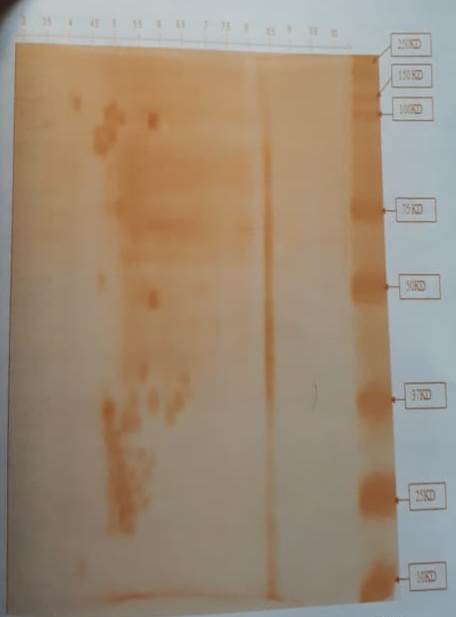


**Fig:** 2D-gel electrophoresis for 2D(Normal conditions) and 2DN(stress conditions)


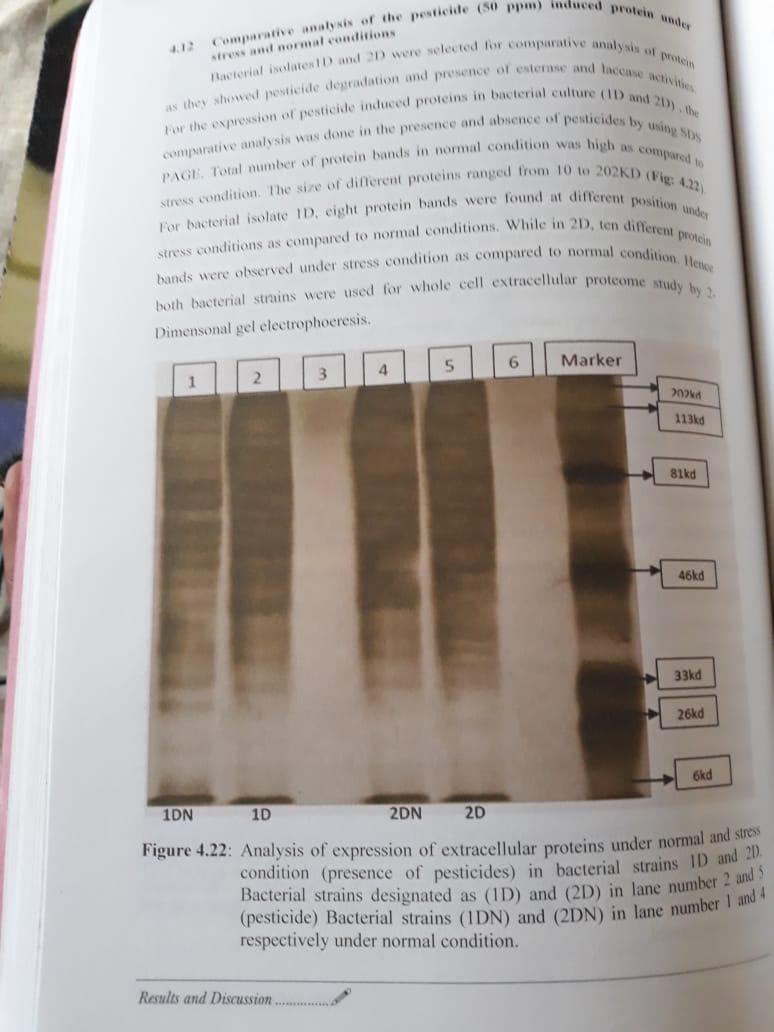


**Fig:** SDS -gel electrophoresis for 2D(Normal conditions) and 2DN(stress conditions)
